# Prevalence of obesity and overweight in African learners: a protocol for systematic review and meta-analysis

**DOI:** 10.1136/bmjopen-2016-013538

**Published:** 2017-01-13

**Authors:** Theodosia Adom, Thandi Puoane, Anniza De Villiers, André Pascal Kengne

**Affiliations:** 1 Nutrition Research Centre, Radiological and Medical Sciences Research Institute, Ghana Atomic Energy Commission, Legon, Ghana; 2 Faculty of Community and Health Sciences, School of Public Health, University of the Western Cape, South Africa; 3 Non-communicable Disease Research Unit, South African Medical Research Council, South Africa; 4 University of Cape Town, South Africa

**Keywords:** EPIDEMIOLOGY, NUTRITION & DIETETICS

## Abstract

**Introduction:**

Obesity and overweight are an emerging problem in Africa. Obese children are at increased risk of developing hypertension, high cholesterol, orthopaedic problems and type 2 diabetes as well as increased risk of adult obesity. Prevention of childhood overweight and obesity therefore needs high priority. The review approach is particularly useful in establishing whether research findings are consistent and can be generalised across populations and settings. This systematic review aims to assess the magnitude and distribution of overweight and obesity among primary school learners within populations in Africa.

**Methods and analysis:**

A comprehensive search of key bibliographic databases including MEDLINE (PubMed), MEDLINE (EbscoHost), CINAHL (EbscoHost), Academic Search Complete (EbscoHost) and ISI Web of Science (Science Citation Index) will be conducted for published literature. Grey literature will be also be obtained. Full-text articles of eligible studies will be obtained and screened following predefined inclusion criteria. The quality of reporting as well as risk of bias of included studies will be assessed, data extracted and synthesised. The results will be summarised and presented by country and major regional groupings. Meta-analysis will be conducted for identical variables across studies. This review will be reported following the MOOSE Guidelines for Meta-Analysis and Systematic Reviews of Observational Studies.

**Ethics and dissemination:**

Ethics is not a requirement since no primary data will be collected. All data that will be presented in this review are based on published articles. The findings of this systematic review will be submitted for publication in peer-reviewed journals and disseminated in national and international conferences and also in policy documents to appropriate bodies for decision-making, where needed. It is expected that the findings will identify some research gaps for further studies.

**Strengths and limitations of this study**

- The use of reference methodologies to guide the study design from study selection to synthesis.
- All eligible studies of all languages will be included.
- The inclusiveness of studies conducted in all countries within the continent.
- Including only studies that used recognised body mass index cut-offs may eliminate some relevant studies that used other measures of body composition.
- The inclusion of only school-based surveys may exclude other relevant studies.

## Introduction

Childhood obesity continues to be a serious public health problem across the globe. The prevalence is increasing to public health proportions among preschool children.^1^ According to estimates by the International Obesity Task Force, 155 million school-going children worldwide are either overweight or obese.^2^ Obesity/overweight is an emerging problem in Africa. In some cultures, women who are fat or ‘rounded’ are considered a sign of wealth, fertility and beauty while lean individuals are considered malnourished or a sign of ill health^3^ ^4^

Overweight and obesity are terms used to describe an excess of adiposity (or fatness) above the ideal for good health. According to the WHO, overweight and obesity are the fifth leading cause of mortality globally and a major risk factor for non-communicable diseases including cardiovascular diseases, diabetes and some cancers.^5^ Obese children are at increased risk of developing hypertension, high cholesterol, orthopaedic problems and type 2 diabetes.^6^ There is also a considerable wealth of evidence that overweight and obesity in childhood present an increased risk of adult obesity^7^ ^8^ with associated health risks in adulthood. In addition to the health consequences of overweight and obesity in childhood, there is some evidence to suggest that weight status may affect school performance in children, which may negatively affect long-term career development.^9^ ^10^ There is, however, limited evidence in some other studies on the negative association between weight status and academic achievement.^11–13^

Prevention of childhood overweight and obesity therefore needs high priority. Schools are effective for implementing behaviour change in children^14^ ^15^ which will have a long-term impact. For effective intervention among learners, however, there is the need for evidence of overweight and obesity prevalence. Some systematic reviews on prevalence combined the findings of children and adolescents/youth.^16^ ^17^ No systematic review has been conducted that involved populations of primary school learners residing in all countries within Africa until now, to support the need for intervention programmes across the continent. Moreover, a more recent attempt among school-aged children in Africa was limited to sub-Saharan Africa.^16^ The current review, to the best of our knowledge, is the first effort to systematically review the existing literature on overweight and obesity among primary school learners in Africa to identify gaps in the literature for further research.

This protocol is developed following the guidelines of PRIMSA-P 2015.^18^

### Objectives

To conduct systematic review and meta-analysis to assess the magnitude and distribution of overweight and obesity among primary school learners aged 6–12 years of both sexes within countries in Africa, using any of the internationally accepted body mass index cut-offs: (Centers for Disease Control and Prevention,^19^ International Obesity Task Force,^20^ WHO).^21^

### Review question

What is the prevalence of overweight and obesity among primary school learners within countries in Africa as reported in studies published between 1980 and 2016?

## Methods

The MOOSE Guidelines for Meta-Analyses and Systematic Reviews of Observational Studies^22^ will guide the methods for this systematic review.

### Inclusion criteria

Studies that will meet the following criteria will be included in the review:

1. Cross-sectional school-based surveys or the cross-sectional evaluation in longitudinal school-based surveys, involving primary school learners aged between 6–12 years of African populations residing in African countries and reporting a prevalence of overweight and obesity.
2. Any objective measure of body composition, that is, body weight, height, body mass index, sum of skinfolds and body fat
3. All published and unpublished studies between 1 January 1980 and 30 June 2016.
4. No language limitations will be set.

### Exclusion criteria

1. Studies that are not school-based
2. Intervention studies
3. Studies conducted in African populations but residing outside Africa.
4. Studies carried out on learners who are suffering from critical illness or with chronic conditions.

### Search strategy

Search strategy will involve a series of complementary search methods including a comprehensive search of key bibliographic databases and manual search of reference lists or citations follow-up of identified eligible articles and relevant reviews which will not be captured through the bibliographic databases search. Using relevant search terms that will be developed from Medical Subject Headings (MeSH), keywords generated from the subject headings and the names of the 54 African countries and the five African subregions (African search filter),^23^ a systematic search of MEDLINE (PubMed), MEDLINE (EbscoHost) CINAHL (EbscoHost), Academic Search Complete (EbscoHost) and ISI Web of Science (Science Citation Index) will be carried out for published literature on overweight and obesity in learners in Africa. Examples of search terms to be used include the following: ‘obese’, ‘obesity’, ‘overweight’, ‘over weight’, ‘over-weight’, ‘weight disorder’, ‘body composition’, ‘body mass index’, ‘body weight’, ‘BMI’, ‘body fat’, ‘adiposity’, ‘percent body fat’, ‘body fat distribution’. These various combinations will be used to suit each database. A search strategy for the PubMed-MEDLINE database is attached (see online supplementary appendix 1).

10.1136/bmjopen-2016-013538.supp1 supplementary appendix

Grey literature (including reports and conference proceedings) will be searched through the Google scholar search engine and key relevant websites such as Opengrey, WHO and African Index Medicus. Key individuals in the field will be contacted for any unpublished work and research papers that are under preparation. References will be exported and duplicates will be removed using citation management software.

### Selection of studies

The titles and abstracts of potentially relevant identified articles will be independently screened by two researchers for eligibility. Full-text copies of articles that will meet the eligibility criteria will be obtained. These full-text articles will then be assessed by two independent researchers for inclusion in the review. Any disagreement about the eligibility will be resolved through discussion with a third assessor. A short questionnaire was developed and will be used to guide the selection of relevant studies (see online supplementary appendix 2).

10.1136/bmjopen-2016-013538.supp2 supplementary appendix

### Quality assessment of included studies

The quality of full-text articles that will be included in the study will be assessed by a modified version of the Downs and Black assessment tool.^24^ All questions on randomised controlled trials and intervention study designs will be eliminated. This checklist provides scores for the quality of reporting, internal validity (bias) and external validity (see online supplementary appendix 3).

10.1136/bmjopen-2016-013538.supp3 supplementary appendix

### Data extraction

This will be carried out independently by two researchers. The following relevant data will be extracted on all included studies using a standardised data form: study details (author, year of publication, year of beginning of study, country of study, type of publications), study characteristics (study design, mean/median age and range, sampling method (random vs non-random), sample size, criteria for classification of overweight and obesity, study setting (urban and rural), type of sample (national vs subnational and local), gender distribution, distribution by location, African region where the study country is located), prevalence of overweight and obesity (overall and by gender and location).

### Data synthesis, assessing heterogeneity and publication bias

Data extracted will be summarised by country and regional prevalence (Central Africa, Eastern Africa, Southern Africa, Northern Africa and Western Africa) and, where possible, by gender and location. Meta-analysis and meta-regression analysis will be conducted for identical variables across studies. Pooled estimates for the meta-analysis and their 95% CIs will be obtained using the random-effects model of DerSimonian-Laird.^25^ Studies will be weighted by the inverse of their variances. To minimise the effect of studies with extreme prevalence rates on pooled estimates, variance stabilisation will be achieved through arc-sine transformation of the estimates before pooling.^26^

Heterogeneity of studies will be assessed with Cochran's Q statistic.^27^ This index is, however, limited in that it gives only the statistical significance but not the true extent of heterogeneity. To determine the degree of heterogeneity between studies, the I^2^ statistic^28^ will be used. I^2^ values of 25%, 50% and 75% would represent mean low, medium and high heterogeneity, respectively.

To assess the potential sources of heterogeneity, subgroup analyses using sex, age, sample size, setting (rural–urban; private–public school), year of beginning of study, criteria for classification of overweight and obesity, and geographical region (Central Africa, Eastern Africa, Southern Africa, Northern Africa and Western Africa) will be performed. Heterogeneity will also be tested by conducting meta-regression analysis. Funnel plots and Egger's test of bias^29^ will be used to assess publication bias. The inter-rater agreement for study inclusion will be assessed using Cohen's κ coefficient.^30^

Data analysis will be performed using the R statistical software (The R Foundation for statistical computing, Vienna, Austria).

### Presenting and reporting the review results

A PRISMA flow chart of search and study selection with included and excluded studies will be presented. Reasons for exclusion of studies will be given. Extracted data will be presented in tables. Summary statistics of quantitative data will be complemented with narrative syntheses. Quantitative data will be presented in tables of individual studies, summary tables and forest plots where appropriate. Prevalence will be examined by country (all 54 countries), region (Central Africa, Eastern Africa, Southern Africa, Northern Africa and Western Africa), sex and settings (urban–rural; private–public school). The quality assessment and risk of bias scores determined for each included study will be presented in tables.

## Conclusion

It is expected that this systematic review will provide relevant evidence on the magnitude and distribution of overweight and obesity in schoolgoing children residing in Africa, thereby supporting the need for appropriate intervention strategies to control the increasing prevalence. It is also expected that more information on the criteria commonly used in African countries to classify overweight and obesity in children will be obtained. This review will hopefully identify some research gaps for further studies.

### Ethics and dissemination

Ethics is not a requirement since no primary data will be collected. All data that will be presented in this review are based on published articles. The findings of this systematic review will be submitted for publication in a peer-reviewed journal. This will also form a chapter of a PhD thesis. In addition, this will be disseminated in conferences and policy documents to appropriate bodies for decision-making where needed. It is expected that this systematic review will provide relevant evidence on the magnitude and distribution of overweight and obesity in schoolchildren residing in Africa, thereby supporting the need for appropriate intervention strategies to control the increasing prevalence. This review will hopefully identify some research gaps for further studies.

### Protocol registration

Details of the protocol for this systematic review were registered on PROSPERO and can be accessed at http://www.crd.york.ac.uk/PROSPERO/display_record.asp?ID=CRD42016035248.
