# Supplementary material for: Prevalence of obesity and overweight in African learners: a protocol for systematic review and meta-analysis
Source: BMJ Open. 2017 Jan 13;7(1):e013538. doi: 10.1136/bmjopen-2016-013538 (PMC5253553; doi:10.1136/bmjopen-2016-013538)
Supplement: supplementary appendix [file bmjopen-2016-013538supp_appendix1.pdf]

Appendix 1: Search strategy PubMed

Search	Search terms	Hits
1	Obesity [tw] OR obese [tw] OR overweight [tw] over weight [tw] OR over-weight [tw] OR weight disorder* [tw] OR body composition [tw] OR body mass index [tw] OR body weight* [tw] OR BMI [tw] OR body fat [tw] OR percent* body fat [tw] OR body fat percent* [tw] OR body fat distribution [tw] OR adiposity [tw] OR skinfold thickness [tw] OR skinfolds [tw]	
2	Obesity [MeSH Terms]	
3	Overweight [MeSH Terms]	
4	# 1 OR # 2 OR # 3	
5	Learners [tw] OR schoolchildren [tw] OR school children [tw] OR school-children [tw] OR school going children [tw] OR school-going children [tw]	
6	# 4 AND # 5	
7	((((("Africa"[MeSH] OR Africa*[tw] OR Algeria[tw] OR Angola[tw] OR Benin[tw] OR Botswana[tw] OR "Burkina Faso"[tw] OR Burundi[tw] OR Cameroon[tw] OR "Canary Islands"[tw] OR "Cape Verde"[tw] OR "Central African Republic"[tw] OR Chad[tw] OR Comoros[tw] OR Congo[tw] OR "Democratic Republic of Congo"[tw] OR Djibouti[tw] OR Egypt[tw] OR "Equatorial Guinea"[tw] OR Eritrea[tw] OR Ethiopia[tw] OR Gabon[tw] OR Gambia[tw] OR Ghana[tw] OR Guinea[tw] OR "Guinea Bissau"[tw] OR "Ivory Coast"[tw] OR "Cote d'Ivoire"[tw] OR Jamahiriya[tw] OR	

	Jamahiryia[tw] OR Kenya[tw] OR Lesotho[tw] OR Liberia[tw] OR Libya[tw] OR Libia[tw] OR Madagascar[tw] OR Malawi[tw] OR Mali[tw] OR Mauritania[tw] OR Mauritius[tw] OR Mayote[tw] OR Morocco[tw] OR Mozambique[tw] OR Mocambique[tw] OR Namibia[tw] OR Niger[tw] OR Nigeria[tw] OR Principe[tw] OR Reunion[tw] OR Rwanda[tw] OR "Sao Tome"[tw] OR Senegal[tw] OR Seychelles[tw] OR "Sierra Leone"[tw] OR Somalia[tw] OR "South Africa"[tw] OR "St Helena"[tw] OR Sudan[tw] OR Swaziland[tw] OR Tanzania[tw] OR Togo[tw] OR Tunisia[tw] OR Uganda[tw] OR "Western Sahara"[tw] OR Zaire[tw] OR Zambia[tw] OR Zimbabwe[tw] OR "Central Africa"[tw] OR "Central African"[tw] OR "West Africa"[tw] OR "West African"[tw] OR "Western Africa"[tw] OR "Western African"[tw] OR "East Africa"[tw] OR "East African"[tw] OR "Eastern Africa"[tw] OR "Eastern African"[tw] OR "North Africa"[tw] OR "North African"[tw] OR "Northern Africa"[tw] OR "Northern African"[tw] OR "South African"[tw] OR "Southern Africa"[tw] OR "Southern African"[tw] OR "sub Saharan Africa"[tw] OR "sub Saharan African"[tw] OR "subSaharan Africa"[tw] OR "subSaharan African"[tw]) NOT ("guinea pig"[tw] OR "guinea pigs"[tw] OR "aspergillus niger"[tw])))))	
8	# 6 AND # 7	
9	# 8 Limits: 1980/01/01 to 2016/06/30	
