# Supplementary material for: Prevalence of obesity and overweight in African learners: a protocol for systematic review and meta-analysis
Source: BMJ Open. 2017 Jan 13;7(1):e013538. doi: 10.1136/bmjopen-2016-013538 (PMC5253553; doi:10.1136/bmjopen-2016-013538)
Supplement: supplementary appendix [file bmjopen-2016-013538supp_appendix2.pdf]

Appendix 2: A short question guide for the selection of relevant studies based on inclusion criteria

No	Question	Action
1	Did the study use any of the eligible study design?	Yes, move to next question<br>No, exclude
2	Did the study involve learners/schoolchildren aged 6-12 years?	Yes, move to next question<br>No, exclude
3	Were the study participants residing in any of the African countries?	Yes, move to next question<br>No, exclude
4	Did the study report overweight and/ obesity?	Yes, move to next question<br>No, exclude
5	Were objective measures of body composition used?	Yes, move to next question<br>No, exclude
6.	Were any of the internationally accepted cut-offs for overweight and obesity used?	Yes, include study<br>No, exclude
