# Supplementary material for: Prevalence of obesity and overweight in African learners: a protocol for systematic review and meta-analysis
Source: BMJ Open. 2017 Jan 13;7(1):e013538. doi: 10.1136/bmjopen-2016-013538 (PMC5253553; doi:10.1136/bmjopen-2016-013538)
Supplement: supplementary appendix [file bmjopen-2016-013538supp_appendix3.pdf]

Appendix 3: Modified Downs and Black checklist for assessing quality of studies

No.	Question	Yes = 1	No = 0
**Reporting**			
1.	Is the hypothesis/aim/objective of the study clearly described?		
2.	Are the main outcomes of the study clearly described?		
3	Are the characteristics of the patients included in the study clearly described?		
6	Are the main findings of the study clearly described?		
7	Does the study provide estimates of the random variability in the data for the main outcomes?		
10	Have actual probability values been reported (e.g. 0.035 rather than <0.05) for the main outcomes except where the probability value is less than 0.001?		
External validity			
11	Were the subjects asked to participate in the study representative of the entire population from which they were recruited?		
12	Were those subjects who were prepared to participate representative of the entire population from which they were recruited?		
Internal validity			
18	Were the statistical tests used to assess the main outcomes appropriate?		
20	Were the main outcome measures used accurate (valid and reliable)?		
